# F-18 FDG PET/CT in 26 patients with SAPHO syndrome: a new vision of clinical and bone scintigraphy correlation

**DOI:** 10.1186/s13018-018-0795-0

**Published:** 2018-05-22

**Authors:** Xiaochuan Sun, Chen Li, Yihan Cao, Ximin Shi, Li Li, Weihong Zhang, Xia Wu, Nan Wu, Hongli Jing, Wen Zhang

**Affiliations:** 1 Department of Rheumatology and Clinical Immunology, Peking Union Medical College Hospital, Peking Union Medical College and Chinese Academy of Medical Sciences, No. 1 Shuaifuyuan, Beijing, 100730 People’s Republic of China; 2 Department of Traditional Chinese Medicine, Peking Union Medical College Hospital, Peking Union Medical College and Chinese Academy of Medical Sciences, No. 1 Shuaifuyuan, Beijing, 100730 People’s Republic of China; 3 Department of Nuclear Medicine, Peking Union Medical College Hospital, Peking Union Medical College and Chinese Academy of Medical Sciences, No. 1 Shuaifuyuan, Beijing, 100730 People’s Republic of China; 4 Department of Dermatology, Peking Union Medical College Hospital, Peking Union Medical College and Chinese Academy of Medical Sciences, No. 1 Shuaifuyuan, Beijing, 100730 People’s Republic of China; 5 Department of Radiology, Peking Union Medical College Hospital, Peking Union Medical College and Chinese Academy of Medical Sciences, No. 1 Shuaifuyuan, Beijing, 100730 People’s Republic of China; 6 Department of Orthopaedics, Peking Union Medical College Hospital, Peking Union Medical College and Chinese Academy of Medical Sciences, No. 1 Shuaifuyuan, Beijing, 100730 People’s Republic of China

**Keywords:** SAPHO, [18F] FDG PET/CT, Bone scintigraphy, Osteoarticular symptoms

## Abstract

**Backgrounds:**

Whole-body bone scintigraphy (WBBS) and MRI are widely used in assessment of patients with synovitis, acne, pustulosis, hyperostosis, and osteitis (SAPHO) syndrome. However, the value of F-18 fluorodeoxyglucose-positron emission tomography/computed tomography (^18^F-FDG PET/CT) in SAPHO syndrome was unclear. The aim of this study was to characterize the manifestation of SAPHO syndrome on ^18^F-FDG PET/CT and explore its relationship with clinical symptoms and WBBS.

**Methods:**

Twenty-six patients who suffered from SAPHO syndrome and had undergone whole-body ^18^F-FDG PET/CT were recruited in Peking Union Medical College Hospital from 2004 to 2016. Clinical manifestations and laboratory findings were recorded for all patients. Imaging data on 18F-FDG PET/CT and WBBS were collected and analyzed retrospectively.

**Results:**

All the 26 patients (20 females and 6 males) exhibited skeletal abnormalities on ^18^F-FDG PET/CT. Multiple skeletal lesions affecting the anterior chest wall or spine with low to moderate ^18^F-FDG uptake and coexistence of osteolysis and osteosclerosis presented as the typical features of SAPHO syndrome. Sixteen (61.5%) patients had abnormal ^18^F-FDG uptake outside the osteoarticular system. PET scan had moderate to substantial agreement with CT and WBBS in revealing lesions in the anterior chest wall and axial skeleton. Nonetheless, the correlation between increased ^18^F-FDG uptake and clinical symptoms was weak.

**Conclusions:**

SAPHO syndrome exhibits characteristic features on ^18^F-FDG PET/CT. It showed comparable capacity in revealing skeletal lesions with bone scintigraphy.

**Electronic supplementary material:**

The online version of this article (10.1186/s13018-018-0795-0) contains supplementary material, which is available to authorized users.

## Background

SAPHO (synovitis, acne, pustulosis, hyperostosis, and osteitis) syndrome is a rare disease which was firstly proposed by Chamot et al. in 1987 as an umbrella term for a spectrum of osteoarticular and dermatological disorders [1]. High heterogeneity has been demonstrated in its clinical manifestations, in which bone disease is a core element [2]. Despite recent advances in our understanding of SAPHO syndrome, the precise pathogenesis largely remains elusive [3]. The diagnosis of SAPHO is based on clinical, radiological, and sometimes histological features, with no standard criteria accepted universally [3].

SAPHO syndrome is considered to be a rare disease, with a prevalence lower than 1/10,000 in Caucasians [4]. However, exact epidemiological data is still unavailable; the real prevalence is probably underestimated due to lack of awareness of this disease and the absence of skin lesions in some patients [5, 6]. Despite the good overall prognosis of most patients, SAPHO appears to be a chronic disease with recurrent exacerbations and remissions, severely undermining patients’ general health and quality of life [6, 7].

Radiological examinations play an important role in differential diagnosis and evaluation of osteoarticular abnormalities in SAPHO syndrome, especially subclinical foci [8]. Currently, whole-body bone scintigraphy and MRI are commonly used, which allows for early diagnosis and higher sensitivity compared with conventional radiographic imaging [3, 9]. The use of F-18 fluorodeoxyglucose-positron emission tomography/computed tomography (^18^F-FDG PET/CT) in SAPHO has been described by several case reports [10–21] (Table 1) and has been demonstrated to be effective in excluding metastatic disease in challenging cases and differentiating active lesions from inactive lesions [22]. However, few studies on the value of PET/CT in SAPHO syndrome have been performed in relatively large population.

**Table 1:** Summary of reported patients with SAPHO syndrome undergoing PET/CT

Author/year	Age	Sex	Skin lesions	Sites of osteoarticular symptoms	PET/CT					Bone scintigraphy	ESR (mm/h)	CRP (mg/dL)
					Time from onset	Sites of increased tracer uptake	SUVmax	Site of lesions on CT	Characteristics of lesions on CT			
Kohlfuerst et al. 2003 [10]	45	M	PPP	Shoulder, tibia^(R)^	< 36	Tibia, ankle^*^	NA	ACW	Hyperostosis	ACW, tibia	NA	NA
Pichler et al. 2003 [11]	48	M	PPP, acne, seborrhiasis	ACW	> 48	ACW	NA	ACW	Sclerosis	ACW	NA	NA
Shibakuki et al. 2006 [12]	57	M	PPP, acne	ACW	~ 240	Normal	NA	CS	Bone defect, consolidation	ACW, CS, rib 6,7	15	Normal
Inoue et al. 2007 [13]	74	F	PPP	Back	300	CS, TS	NA	CS, TS, LS	Sclerosis, syndesmophyte, osteophyte formation	CS, TS, LS, SI	Elevated	Elevated
Takeuchi et al. 2007 [14]	50	F	None	Neck, back, lumbus	168	ACW, CS, LS, SI, wrist	1.6	CS, LS, femoral neck	Osteosclerosis, osteolysis	ACW, CS, LS, SI, femoral neck	87	2.5
Patel et al. 2009 [15]	20	F	PPP	Back	N/A	ACW, TS, SI	NA	NA	NA	SC, TS, SI	NA	NA
Abuhid et al. 2010 [16]	44	F	PPP	ACW, SI	36	ACW, SI	2.18	ACW, SI	Sclerosis, irregularities	ACW, SI	NA	NA
Canbaz et al. 2010 [17]	18	F	None	Diffuse, prominent in back	9	TS, LS, pelvis	NA	NA	NA	Spine, pelvic bones	74	NA
Nakamura et al. 2010 [18]	60	F	None	Ankle^(Bi)^, back	N/A	CS, LS, SI^(L)^, shoulder^(L)^	6.1	NA	NA	NA	99	6.379
Namkoong et al. 2015 [19]	62	M	None	ACW^(R)^	3	ACW	4.0	None	Normal	NA	NA	13.45
Ikeda et al. 2015 [20]	60	F	Acne	ACW, hands^(Bi)^	N/A	ACW	NA	ACW	Hyperostosis	NA	NA	NA
Dong et al. 2016 [21]	60	F	PPP	Back	2	TS, SS	NA	TS, SS	Osteolysis	TS, S1	NA	NA
Dong et al. 2016 [21]	51	F	None	Back	7	ACW, LS, SI	NA	ACW, SI	Osteosclerosis	ACW, spine, SI	NA	NA

ACW includes the costochondral, sternoclavicular, manubriosternal, and costosternal articulation, sternum, clavicles, and anterior ribs

*ACW* anterior chest wall, *SI* sacroiliac, *CS* cervical spine, *TS* thoracic spine, *LS* lumbar spine, *NA* not applicable

^*^With absent uptake in the left SC region

The aim of this study was to characterize the manifestation of SAPHO syndrome on ^18^F-FDG PET/CT in 26 patients and explore its relationship with clinical symptoms and whole-body bone scintigraphy.

## Methods

### Study population

Twenty-six patients who fulfilled the diagnostic criteria for SAPHO syndrome proposed by Benhamou et al. [4] and had undergone whole-body ^18^F-FDG PET/CT after 6 months prior to the onset of symptoms were recruited in Peking Union Medical College Hospital (PUMCH) from 2004 to 2016. Inclusion criteria included osteoarticular manifestations with acne conglobate, acne fulminans or hidradenitis suppurativa, osteoarticular manifestations with palmoplantar pustulosis (PPP), hyperostosis (of the anterior chest wall (ACW), limbs, or spine) with or without dermatosis, and CRMO involving the axial or peripheral skeleton with or without dermatosis. Exclusion criteria included septic osteomyelitis, infectious chest wall arthritis, and infectious PPP, palmoplantar keratodermia, and diffuse idiopathic skeletal hyperostosis except for fortuitous association and osteoarticular manifestations of retinoid therapy. A written informed consent was obtained from each patient. The Ethics Committee of Peking Union Medical College Hospital, Peking Union Medical College and Chinese Academy of Medical Sciences, approved this study (number of Ethics documents: ZS-944).

### Clinical evaluation

All patients’ medical data were collected, including age, sex, skin lesions, sites of osteoarticular symptoms, and time interval between onset of SAPHO-related symptoms and PET/CT examination. Laboratory tests included erythrocyte sedimentation rate (ESR), C-reactive protein (CRP), anti-nuclear antibody (ANA), rheumatoid factor (RF), and HLA-B27. ESR and CRP were recorded in the same episode of disease when PET/CT was performed. One episode of disease was defined as a period of time shorter than 2 months, in which no changes of patients’ conditions were reported, including the site, quality, and intensity of pain, and no medications were administrated except for non-steroidal anti-inflammatory drugs (NSAIDs). Some patients had undergone tissue biopsy to aid the diagnosis. Their pathological characteristics were also collected.

### Imaging assessment

Whole-body ^18^F-FDG PET/CT and ^99m^Tc static whole-body bone scintigraphy (WBBS) available on recruitment were collected for all patients. When reporting bone lesions, the costochondral, sternoclavicular, manubriosternal, and costosternal joints, sternum, clavicles, and anterior ribs were grouped as anterior chest wall (ACW). For standard uptake value (SUV) of PET/CT in each patient, the highest one (SUVmax) among all the sites of bone lesions or extra-osteoarticular lesions was recorded separately.

### Statistical analysis

Continuous data were expressed as means ± standard deviation, while categorical variables were described in numbers and percentages. Agreement between functional part of PET/CT and concurrent clinical symptoms, WBBS, and the structural part of PET/CT were assessed using cross-tabulation quantified by Cohen’s *κ*, positive agreement, and negative agreement [23]. Each anatomical region (the ACW, the cervical, thoracic, lumbar, and sacral spine, and the sacroiliac joint) was analyzed separately. Peripheral lesions were not included in agreement assessment due to their low frequency. All tests were two-tailed with the significant level of 0.05. Data were analyzed using SPSS 15.0.

## Results

### Demographic and clinical characteristics

Twenty women and six men were included in the study. The mean age at onset was 44.6 years, while the mean age of performing PET/CT scans was 47.9 years. Almost all patients had both osteoarticular and cutaneous symptoms. Only one (4%) patient showed no sign of skin lesions. The most common dermatological manifestation was palmoplantar pustulosis (PPP), affecting 23 (89%) patients. Severe acne (SV) and psoriasis vulgaris (PV) were seen in 4 (15%) and 2 (8%) patients, respectively. All patients suffered from pain in ACW. Other common sites of osteoarticular symptoms were the pelvis, shoulder, back, hip, and neck, showing an obvious predilection for axial bone and joints.

Laboratory findings were considerably variable among patients, with ESR ranging from 3 to 115 mm/h and CRP from 2 to 120 mg/dl. Over 60% patients had both an abnormal ESR (> 20 mm/h) and an abnormal CRP (> 5 mg/dl). No patients were found to be HLA-B27 positive. Nine patients underwent bone biopsies in order to exclude infectious or metastatic diseases. All biopsies were performed right after PET/CT examination, and the site of sampling was selected as the site with highest SUV. The most frequent finding was aseptic inflammation with infiltration of inflammatory cells, typically including lymphocytes and plasma cells. Lesions with subacute inflammation, chronic inflammation, and fibrosis and hyperostosis were revealed in different patients (Additional file 1: Table S1).

### Whole-body bone scintigraphy

Twenty-five (96%) patients underwent ^99m^Tc-MDP whole-body bone scintigraphy. All had increased tracer uptake in ACW. Twenty-two (88%) patients had spinal lesions detected by bone scintigraphy, including 15 (60%), 11 (44%), 4 (16%), and 3 (12%) patients for lumbar, thoracic, cervical, and sacral spine, respectively. Additionally, 8 (32%) patients had abnormal uptake in sacroiliac joint and 9 (36%) in non-axial bones.

### ^18^F-FDG PET/CT

**Fig. 1:**
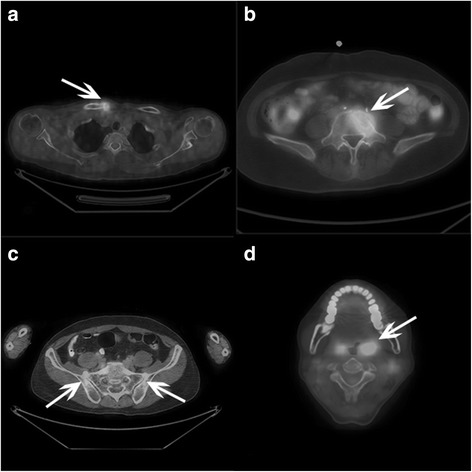
Lesions revealed by ^18^F-FDG PET/CT in SAPHO syndrome (white arrows). Abnormally increased uptake of ^18^F-FDG can be seen in **a** right sternoclavicular joint, **b** first lumbar vertebra, **c** bilateral sacroiliac joints, and **d** left tonsil

All the 26 patients exhibited skeletal abnormalities on ^18^F-FDG PET, of which 22 (84.6%) showed multiple bone lesions on PET imaging. The ACW was the most frequently involved site with 23 (88.5%) patients affected (Fig. 1a). Increased ^18^F-FDG uptake in lumbar, thoracic, cervical, and sacral spine were found in 4 (15.4%), 11 (42.3%), 15 (57.7%), and 5 (19.2%) patients, respectively (Fig. 1b). Lesions of pelvis were detected in 9 (34.6%) patients, with 7 (26.9%) and 4 (15.4%) patients having lesions on sacroiliac joint (Fig. 1c) and ilium, respectively. Four (15.4%) patients showed abnormal ^18^F-FDG uptake located in other non-axial sites. The SUVmax of the skeletal lesions ranged from 1.2 to 17.1, with a majority (92.3%) of patients exhibit low to moderate ^18^F-FDG uptake, yet one patient exhibits intense uptake (SUVmax 13.1) and one very intense uptake (SUVmax 17.1). The structural part of PET/CT demonstrated features of both acute inflammation, such as osteolysis and soft tissue edema, and chronic inflammation, osteosclerosis, and hyperostosis (Table 2 and Additional file 1: Table S1).

**Table 2:** Demographic, clinical, and imaging characteristics of the 26 patients with SAPHO syndrome

Age at onset (years)	44.6 ± 11.1	SS	5 (19.2%)
Gender, female	20 (74.1%)	Ilium	4 (15.4%)
Age at performing PET/CT, years	47.9 ± 10.8	CS	4 (15.4%)
Skin lesions	25 (96.2)	Phalanx	2 (7.7%)
PPP	23 (88.5%)	Shoulder	1 (3.8%)
SA	4 (15.4%)	Femur	1 (3.8%)
PV	2 (7.7%)	SUVmax	6.0 ± 3.4
Osteoarticular symptoms	26 (100%)	Site of lesions on CT	
Anterior chest pain	26 (100%)	ACW	17 (65.4%)
Cervical region pain	6 (23.1%)	LS	13 (50.0%)
Thoracic region pain	12 (46.2%)	TS	10 (38.5%)
Lumbosacral region pain	21 (80.8%)	SI	6 (23.1%)
Hip pain	9 (34.6%)	SS	6 (23.1%)
Others	6 (19.2%)	Ilium	4 (15.4%)
Laboratory findings		CS	4 (15.4%)
ESR, mm/h	46.9 ± 8.2	Phalanx	3 (11.5%)
CRP, mg/L	22.4 ± 7.9	Posterior rib	1 (3.8%)
HLA-B27, positive	0 (0%)	Characteristics of lesions on CT	
Bone scintigraphy	25	Osteolysis	16 (61.5%)
ACW	25 (100%)	Osteosclerosis	10 (38.5%)
LS	16 (61.5%)	Hyperostosis	8 (30.8%)
TS	12 (46.2%)	Soft tissue edema	5 (19.2%)
SI	8 (32.0%)	Joint space widening	3 (11.5%)
CS	4 (16.0%)	Osteophyte	2 (7.7%)
SS	3 (12.0%)	Increased BMD	2 (7.7%)
Femur	2 (8.0%)	Heterogeneous BMD	2 (7.7%)
Ilium	2 (8.0%)	Decreased BMD	2 (7.7%)
Knee	2 (8.0%)	Increased and decreased BMD	1 (3.8%)
Other	5 (19.2%)	Others	3 (11.5%)
PET/CT		Extra-osteoarticular abnormalities	19 (73.1%)
Osteoarticular abnormalities	26 (100%)	Tonsil, pharynx, nose, paranasal sinus	10 (38.5%)
Sites of increased tracer uptake		Lymph node	8 (30.8%)
ACW	23 (88.5%)	Thyroid gland	5 (19.2%)
LS	15 (57.7%)	Gastrointestinal tract	4 (15.4%)
TS	11 (42.3%)	Lung and pleura	2 (7.7%)
SI	7 (26.9%)	Others	1 (3.8%)

ACW includes the costochondral, sternoclavicular, manubriosternal, and costosternal articulation, sternum, clavicle, and anterior ribs

*ACW* anterior chest wall, *SI* sacroiliac, *CS* cervical spine, *TS* thoracic spine, *LS* lumbar spine, *SS* sacral spine, *BMD* bone marrow density

**Fig. 2:**
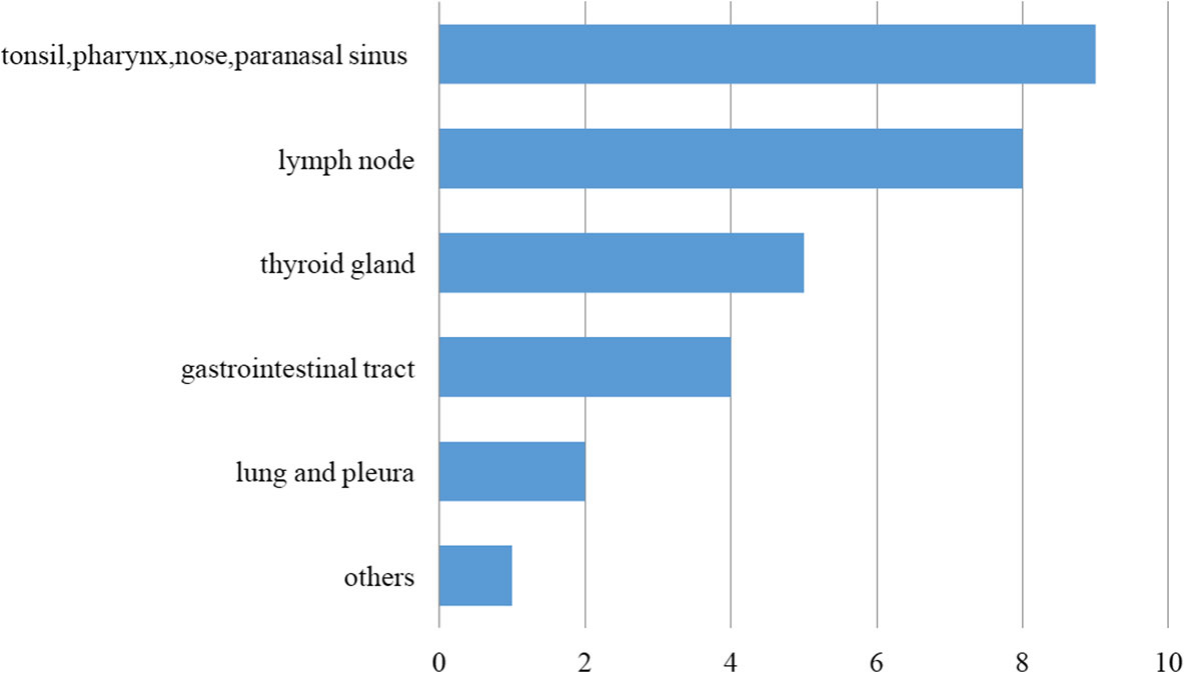
Extra-osteoarticular organs/tissues with abnormal tracer uptake under ^18^F-FDG PET/CT in 26 patients with SAPHO syndrome

Sixteen (61.5%) patients had abnormal ^18^F-FDG uptake outside the osteoarticular system (Fig. 2). The tonsil (Fig. 1d), pharynx, nose, paranasal sinus, and lymph nodes were the most common affected sites, with a combined percentage of 34.6%. Five (19.2%) patients showed abnormal ^18^F-FDG uptake in the thyroid and 4 (15.4%) in the gastrointestinal tract.

### Agreement between PET/CT, WBBS, and clinical symptoms

The agreement between functional and structural parts of PET/CT was substantial in revealing lesions in the cervical spine (*κ* = 0.75), thoracic spine (*κ* = 0.77), sacral spine (*κ* = 0.79), and sacroiliac joint (*κ* = 0.80). Moderate agreement was reached for the lumbar spine (*κ* = 0.53) and ACW (*κ* = 0.45).

As to the clinical symptoms, ^18^F-FDG PET showed only fair agreement with concurrent symptoms for lesions in the ACW (*κ* = 0.24) and thoracic spine (*κ* = 0.30); 75.0 and 34.8% of the asymptomatic patients in these regions showed increased ^18^F-FDG uptake, respectively. Even more slight agreement was found for lesions in the sacroiliac joint (*κ* = 0.17)—a majority (63.6%) of patients with lumbosacral symptoms did not show increased ^18^F-FDG uptake in the sacroiliac joint. On the other hand, moderate agreement was shown for lesions in the cervical spine (*κ* = 0.51), lumbar spine (*κ* = 0.55), and sacral spine (*κ* = 0.49).

In terms of the correlation between ^18^F-FDG PET and WBBS, moderate to substantial agreement was reached for spinal lesions: cervical spine (*κ* = 0.70), thoracic spine (*κ* = 0.77), lumbar spine (*κ* = 0.44), and sacral spine (*κ* = 0.42). Nonetheless, only fair agreement was shown for sacroiliac joint (*κ* = 0.35). The *κ* coefficient was rather low (*κ* = − 0.06) for lesions in the ACW due to substantial imbalance in the table’s marginal totals [24], yet the positive agreement was as high as 91.7% (Table 3).

**Table 3:** Agreement between PET/CT, whole-body bone scintigraphy, and clinical symptoms

			PET/CT functional part		*κ*	95% CI	Negative agreement (%)	Positive agreement (%)
			−	+				
Anterior chest wall	Symptom	−	2	6	0.24	− 0.14–0.61	36.4	82.9
		+	1	17				
	WBBS	−	0	1	− 0.06	− 0.16–0.03	0	91.7
		+	3	22				
	PET/CT structural part	−	3	5	0.45	0.09–0.81	54.5	87.8
		+	0	18				
Cervical spine	Symptom	−	21	2	0.51	0.02–0.99	93.3	57.1
		+	1	2				
	WBBS	−	21	1	0.70	0.32–1.00	95.5	75.0
		+	1	3				
	PET/CT structural part	−	20	0	0.75	0.44–1.00	95.2	80.0
		+	2	4				
Thoracic spine	Symptom	−	15	8	0.30	0.01–0.59	78.9	42.9
		+	0	3				
	WBBS	−	13	1	0.77	0.52–1.00	89.7	87.0
		+	2	10				
	PET/CT structural part	−	13	1	0.77	0.52–1.00	89.7	87.0
		+	2	10				
Lumbar spine	Symptom	–	10	5	0.55	0.25–0.85	76.9	76.9
		+	1	10				
	WBBS	−	7	3	0.44	0.09–0.79	66.7	77.4
		+	4	12				
	PET/CT structural part	−	8	3	0.53	0.20–0.86	72.7	80.0
		+	3	12				
Sacral spine	Symptom	−	15	0	0.49	0.18–0.80	83.3	62.5
		+	6	5				
	WBBS	−	20	3	0.42	− 0.05–0.88	90.9	50.0
		+	1	2				
	PET/CT structural part	−	19	0	0.79	0.51–1.00	95.0	83.3
		+	2	5				
Sacroiliac joint	Symptom	−	12	3	0.17	− 0.19–0.54	70.6	44.4
		+	7	4				
	WBBS	−	15	3	·	− 0.05–0.74	81.1	53.3
		+	4	4				
	PET/CT structural part	−	18	1	0.80	0.55–1.00	94.7	85.7
		+	1	6				

*WBBS* whole-body bone scintigraphy

## Discussion

This was the largest study investigating the characteristics of SAPHO syndrome on ^18^F-FDG PET/CT. We found multiple skeletal lesions affecting the ACW or spine with low to moderate ^18^F-FDG uptake and coexistence of osteolysis and osteosclerosis as typical features of SAPHO syndrome. PET imaging had moderate to substantial agreement with CT and WBBS in revealing the ACW and axial skeletal lesions in SAPHO syndrome. Nonetheless, the correlation between increased ^18^F-FDG uptake and clinical symptoms was weak.

As to the clinical picture, we found that SAPHO syndrome mainly affected middle-aged females, in accordance with previous studies [7, 25, 26]. The ACW was the main target skeletal site, followed by spine and sacroiliac joints, consistent with the disease’s predilection for axial skeleton [3, 27]. Only one patient was free of dermatological symptoms, less than other cohorts [7, 25–27] but similar to our cohort reported previously [28]. PPP was the most common skin manifestation, followed by SV and PV. In line with current literature, SAPHO syndrome presented no association with HLA-B27, which was distinct from seronegative spondyloanthropathy [25, 28].

The characteristics of SAPHO syndrome on ^18^F-FDG PET/CT had only been described in limited case reports (Table 1). Our results showed most patients had multiple skeletal abnormalities. The ACW was the most commonly affected site on both functional and structural part of PET/CT, followed by the lumbar spine, thoracic spine, and sacroiliac joints. Such findings conformed with the results of WBBS and our large cohort study of 164 patients [28], but slightly differed from some reports that thoracic vertebrae were the most frequently involved segment of the spine [29]. In agreement with previous studies, the bone lesions were usually with low to moderate ^18^F-FDG uptake, yet very intense uptake (SUV > 15) could also present in rare cases. The coexistence of osteolysis and osteosclerosis were the major structural changes revealed by CT [9], suggesting a bone remodeling process in SAPHO syndrome [30].

We found that ^18^F-FDG PET imaging had moderate to substantial agreement with ^99m^Tc-MDP WBBS in revealing spinal lesions, despite of asynchrony of the two examinations. This might suggest a coupled abnormal glucose and diphosphonate metabolism in these regions. Such findings were also backed up by the good agreement between PET imaging and CT for axial skeletal lesions. Infiltration of inflammatory cells consuming excessive glucose causes increased local blood flow and pathological bone metabolism which finally results in structural changes [31]. Overall, ^18^F-FDG PET/CT exhibited comparable ability with WBBS in detecting osteoarticular lesions in SAPHO syndrome, though it had much higher spatial resolution.

In contrast, we found poor agreement between PET imaging and clinical symptoms. This might be partly explained by the existence of abundant subclinical lesions in SAPHO syndrome, especially for the ACW and spine [3, 9, 22]. Moreover, the lack of specificity of symptoms in the lumbosacral region might also contribute to the low agreement. On the other hand, it should also be noted that there exist a small number of symptomatic lesions that were negative on PET imaging. We hypothesized that these symptoms might be caused by remnant structural changes of inactive lesions, such as those causing nerve compression [32, 33].

Various stages of bone lesions have been reported in SAPHO syndrome, including acute inflammation, chronic inflammation, and a healing process with osteosclerosis and bone marrow fibrosis [26]. It has been considered that PET/CT is capable of differentiating active bone lesions from inactive ones—the chronic healing lesions present with normal ^18^F-FDG uptake [3, 13, 22]. However, our findings cast doubt on such a viewpoint since biopsy of lesions with augmented ^18^F-FDG uptake sometimes showed fibrosis or sclerosis. Considering that aspiration biopsy might not represent the whole picture of the involved regions, it is hard to tell whether PET/CT can actually reveal inactive lesions and further investigation is needed.

Extra-osteoarticular abnormalities were revealed in more than 60% patients by PET/CT, which had seldom been reported previously. Although SAPHO syndrome is defined as a disorder of bone and skin, whole-body PET/CT allowed us to look into the disease in a systemic picture. Tonsillitis has been reported to be related to the onset and exacerbation of PPP [34, 35]. In a case report by Shiraishi et al., a patient with SAPHO syndrome and hypertrophic pachymeningitis showed complete remission of both diseases after bilateral tonsillectomy [36]. Our findings provided new data for the hypothesis that SAPHO syndrome might be related to focal inflammation such as tonsillitis and sinusitis. We also discovered thyroid abnormalities on PET/CT in around 1/5 patients. The prevalence of antithyroid antibodies has been reported to be as high as 28% in an Italian cohort of SAPHO syndrome [25], but considerably lower (~ 3%) in other studies [37, 38]. Our findings again raised the concern for thyroid disorder in SAPHO syndrome.

Generally, PET scan is considered to be a safe procedure with no known long-term adverse effects. Some potential risks include bleeding and allergic reactions, which are extremely rare. However, the high cost is a major disadvantage and currently limiting its clinical utility.

There were several limitations to this study. First, the imaging assessments were performed in multiple centers, which may cause bias in imaging collection and interpretation. However, as ^18^F-FDG PET/CT is not a routine test for this rare disease—it is usually used for difficult differential diagnosis in clinical practice, it is rather hard to collect data in a single center. Further studies with rigorous imaging assessment are in need. Second, the ^18^F-FDG PET/CT and WBBS were not performed synchronously, which lead to inaccuracy when analyzing agreement. Further gathering of data is needed to support such analysis.

## Conclusions

In summary, SAPHO syndrome exhibits characteristic features on ^18^F-FDG PET/CT. It showed comparable capacity in revealing skeletal lesions with bone scintigraphy. However, the correlation between increased ^18^F-FDG uptake and clinical symptoms was weak.

## Additional file

Additional file 1: **Table S1.** Clinical characteristics, imaging features and pathological findings of 26 patients with SAPHO syndrome. (DOC 84 kb)
