# Supplementary material for: F-18 FDG PET/CT in 26 patients with SAPHO syndrome: a new vision of clinical and bone scintigraphy correlation
Source: J Orthop Surg Res. 2018 May 22;13:120. doi: 10.1186/s13018-018-0795-0 (PMC5964683; doi:10.1186/s13018-018-0795-0)
Supplement: Supplementary file 1 — Table S1. Clinical characteristics, imaging features and pathological findings of 26 patients with SAPHO syndrome. (DOC 84 kb) [file 13018_2018_795_MOESM1_ESM.doc]

**Table S1: Clinical characteristics, imaging features and pathological findings of 26** patients with SAPHO syndrome

| No. | Sex | Age at onset (year) | Skin lesions | Sites of osteoarticular symptoms | WBBS | PET/CT | | | | | Pathological findings | ESR (mm/h) | CRP (mg/dl) |
| --- | --- | --- | --- | --- | --- | --- | --- | --- | --- | --- | --- | --- | --- |
|  |  |  |  |  |  | Time from onset (month) | Sites of increased tracer uptake | SUVmax | Site of lesions on CT | Characteristics of lesions on CT |  |  |  |
| 1 | F | 40 | PPP | ACW^*^, neck, lumbosacral | ACW, CS, LS | 6 | ACW, CS, LS | 3.9 | ACW, CS, LS | Osteolysis, osteosclerosis | (CS^*^) Chronic inflammation | 64 | NA |
| 2 | F | 56 | None | ACW^*^, neck, lumbosacral, hip | ACW, LS, SI, femur | ~120 | ACW, femur | 3.5 | Femur | Hyperostosis | (clavicle^*^) Chronic inflammation | 74 | 2.71 |
| 3 | F | 31 | PPP, SA | ACW^*^ | ACW | 19 | ACW | 13.1 | ACW, TS, LS, ilium | Osteolysis, hyperostosis | (sternum^*^) Non-bacterial osteomyelitis | 57 | 42.8 |
| 4 | M | 33 | PPP, SA | ACW^*^, lumbosacral^*^ | ACW, LS | ~108 | ACW, LS | 5.2 | ACW, LS | Osteolysis, ossification | (sternum, clavicle^*^) Subacute inflammation | 40 | 7.1 |
| 5 | F | 49 | PPP | ACW^*^, back | ACW, TS, knee, ankle | 3 | ACW, TS | 4.7 | TS | Osteosclerosis, heterogeneity of BMD | NA | 17 | 6.79 |
| 6 | F | 52 | PPP | ACW^*^, neck^*^, lumbosacral^*^, hip | ^*^ACW, LS, SI | 5 | ACW, CS, LS, SS, SI | 3.8 | ACW, CS, LS, SS, SI | Abnormal BMD | (L5^*^) Hyperostosis and ossification of cartilage | 51 | 12.3 |
| 7 | F | 23 | PPP | ACW^*^, lumbosacral^*^, hip^*^ | ACW, LS, SI | 6 | ACW, LS, SS | 9.8 | ACW, LS, SS, SI | Osteolysis, osteosclerosis | (sternum^*^) Chronic inflammation | NA | NA |
| 8 | F | 51 | PPP | ACW, back | ACW, TS, LS | -5^†^ | ACW | 2.61 | CS, TS, LS, SS | Hyperostosis | NA | NA | NA |
| 9 | F | 61 | PPP | ACW^*^, lumbosacral^*^ | ACW, CS, TS, LS, SS | 4 | ACW, LS, SI | 8.5 | ACW, LS, SI | Osteolysis, osteosclerosis | NA | 36 | 3.27 |
| 10 | M | 39 | PPP | ACW^*^, back, hip^*^ | ACW, TS, SI | 58 | ACW, TS, LS, ilium | 6.5 | ACW, CS, TS, LS, SS, ilium | Osteolysis, osteosclerosis, hyperostosis | NA | 39 | 9.97 |
| 11 | M | 40 | PPP, SA | ACW, shoulder, lumbosacral^*^ | ^*^ACW, shoulder, LS | 7 | ACW | 4.7 | LS | Excavated lesion | NA | NA | NA |
| 12 | F | 52 | PPP | ACW, back^*^, lumbosacral | ACW, TS, LS | 4 | TS | 3.8 | TS, phalanx | Hyperostosis, soft tissue thickening | NA | NA | NA |
| 13 | F | 54 | PPP | ACW^*^, lumbosacral^*^, hip^*^ | ACW, LS, SS, SI | 3 | ACW, shoulder, LS, SS, SI | 5.6 | ACW, SS, SI | Osteolysis, osteosclerosis, hyperostosis | NA | 44 | 12.3 |
| 14 | M | 37 | PPP | ACW^*^, wrist, back^*^, lumbosacral^*^, hip^*^ | ACW, TS, maxillary | 81 | ACW, TS, LS, phalanx | 3.6 | ACW, phalanx | Hyperostosis, osteosclerosis, decreased BMD | NA | 3 | 3.7 |
| 15 | F | 49 | PPP | ACW, back, lumbosacral^*^ | ACW, TS, LS | ~120 | TS, LS | 7.6 | TS, LS | Osteolysis, hyperostosis | (L5^*^) Necrosis, fibroblastic proliferation | NA | 18.2 |
| 16 | F | 48 | PPP | ACW^*^, back |  | 14 | ACW, TS | 8.8 | ACW, TS | Osteolysis, soft tissue thickening | (sternum^*^) Inflammatory lesion | NA | 5.09 |
| 17 | M | 15 | SA | ACW^*^, lumbosacral, hip^*^ | ACW, SI | ~36 | ACW, SI, ilium | 1.2 | ACW | Joint space widening, osteolysis, osteosclerosis, decreased BMD | NA | 5 | 3.47 |
| 18 | F | 37 | PPP | ACW^*^, neck^*^, lumbosacral, hip, knee^*^ | ACW, ilium | 6 | ACW, ilium, SI | 4.15 | ACW, SI, ilium | Osteolysis | NA | 83 | 53.01 |
| 19 | F | 48 | PPP | ACW^*^, back, lumbosacral | ACW, TS, LS | 39 | ACW, TS, LS | 6.2 | ACW, TS, LS | Increased BMD, edge roughness, soft tissue edema, hyperostosis, nodular low density foci | NA | NA | NA |
| 20 | F | 55 | PPP | ACW^*^, back, lumbosacral, hip^*^, knee^*^ | ^*^ACW, CS ,TS, femur | 6 | CS, TS, SI | 5.88 | CS, TS, SI | Osteolysis, joint space widening, heterogeneous BMD | (CS^*^) No abnormality | NA | NA |
| 21 | M | 50 | PPP | ACW, neck^*^, back | ACW, CS | 2 | ACW, CS | 5.7 | CS | Osteophyte, osteolysis | NA | 29 | 2.11 |
| 22 | F | 42 | PPP | ACW^*^, back, lumbosacral^*^, hip | ACW, TS, LS | 24 | ACW, TS, LS | 3.3 | ACW, TS, LS | Heterogeneous BMD | NA | NA | NA |
| 23 | F | 35 | PPP, PV | ACW, back^*^, lumbosacral^*^ | ACW, TS, SS, SI | ~240 | ACW, TS, LS, SS, SI | 3.7 | ACW, TS, LS, SS, SI | Osteolysis, joint space widening, increased BMD | NA | NA | 55.86 |
| 24 | F | 56 | PPP | ACW, lumbosacral, hip | ACW, LS, SI, knee | 5 | ACW, LS | 5.51 | ACW, LS | Osteolysis, osteosclerosis, soft tissue thickening, osteophyte | NA | NA | NA |
| 25 | F | 57 | PV | ACW^*^, back, lumbosacral | ACW, posterior rib, TS, LS, ilium, foot | 2 | ACW, TS, LS, ilium, phalanx | 17.l | ACW, posterior rib, TS, ilium, phalanx | Osteolysis, osteosclerosis | NA | 115 | 119.54 |
| 26 | F | 49 | PPP | ACW, neck, lumbosacral^*^ | ACW, LS, nasal bone | 2 | ACW, LS, SS | 8.3 | ACW, LS, SS | Increased and decreased BMD, soft tissue edema | NA | NA | NA |

WBBS, whole body bone scintigraphy; ACW, anterior chest wall; SI, sacroiliac; CS, cervical spine; TS, thoracic spine; LS, lumbar spine; SS, sacral spine; BMD, bone marrow density.

ACW includes the costochondral, sternoclavicular, manubriosternal, costosternal articulation, sternum, clavicle and anterior ribs.

^*^ The symptoms appeared, or the bone scintigraphy or biopsy was performed in the same episode of disease with 18F-FDG PET/CT imaging.

^† 18^F-PET/CT performed prior to the onset of symptoms of SAPHO syndrome.
